# Editorial: Environmental Engagement and Cultural Value: Global Perspectives for Protecting the Natural World

**DOI:** 10.3389/fpsyg.2019.02853

**Published:** 2019-12-20

**Authors:** Fanli Jia, Tobias Krettenauer

**Affiliations:** ^1^Department of Psychology, Seton Hall University, South Orange, NJ, United States; ^2^Department of Psychology, Wilfrid Laurier University, Waterloo, ON, Canada

**Keywords:** culture, environmental behavior, value, cross-culture, environmental belief, environmental engagement

Environmental issues have become a focal point in today's global discussion. As a species, humans have now realized the need for environmental engagement. This engagement is intended to heighten awareness about environmental problems, build knowledge through education about the impact of human behaviors on nature, and change human behaviors to increase sustainability. In particular, globalization and migration have led to a growing need to understand environmental engagement across nations and cultures. The way members of a society relate to the environment seems to be culturally patterned, which means that environmental engagement may differ from one culture to another; however, very little research has examined cultural factors in pro-environmental engagement. Motivations, levels of intensity, and forms of pro-environmental behavior can differ dramatically across cultures, contexts of economic development, political systems, and geographic regions (Gifford and Nilsson, 2014; Eom et al., 2016). This special topic in *Frontiers in Psychology* provides researchers with global and diverse perspectives on their theoretical, empirical, and methodological approaches to understand the human-environment interaction in environmental psychology today. The special issue includes four topics: (1) values, norms, and diversity; (2) emotional connectedness with nature; (3) environmental commons, biodiversity, and education; and (4) public perception of local environmental issues.

## Values, Norms, and Diversity

Xiang et al. investigated cultural orientations (individualistic-collectivistic differences) and the role of perceived intractability to individual climate action in a sample of Chinese college students. Through three studies, the authors found a negative association and impact of perceived intractability to act to reduce climate change. Individuals with more individualistic orientations had a greater likelihood for climate change inaction compared to individuals with collectivistic orientations. The authors suggested that policymakers should encourage the public to believe their individual actions are necessary for a collective effort to fight against climate change.

Zhao et al. conducted three studies to explore the relationship between awe, social dominance orientation, and ecological behavior in a sample of Chinese college students. The authors found that the trait tendency of experiencing awe partially enhanced individuals' ecological behaviors because awe reduced their views of human dominance over nature.

Medina et al. postulated that the existing literature presented a conflicting representation of ethnic groups' concerns toward the environment. The authors argued that one possible solution is to investigate both individual and in-group social norms among ethnic minorities' environmental actions. The authors advocated for a holistic approach that evaluates social, cultural, economic, and political influences as well as uses a mixed-method methodology on future research directions.

## Nature Relatedness and Emotional Connectedness with Nature

Marczak and Sorokowski investigated the relationship between emotional connectedness with nature and modernization in the Meru people of Kenya. The authors found that people in this Indigenous non-Western society have overall positive feelings toward nature (generally supporting the biophilia hypothesis). However, levels of modernization in the sample influenced participants' emotional bonds with nature. Participants with a traditional lifestyle (e.g., remote villages in the bush) showed less emotional affinity toward the natural environment than town-dwellers and participants who live near a market town. Furthermore, the authors suggested that individuals in modern societies who only spend time outdoors occasionally might be more inclined to perceive nature in a positive way. In contrast, inhabitants in Meru bush might not feel as connected to nature because they face extremely harsh living conditions every day.

Dornhoff et al. compared nature relatedness (e.g., cognitive, affective, and experiential connection with nature) of high school students from Ecuador and Germany. The authors found Ecuadorian youth rated higher in relatedness to nature and environmental concern. This cultural difference was reflected in country-specific differences in the structure of environmental concern. Time spent in nature and self-transcendent values positively predicted nature relatedness and environmental concern in both samples.

## Environmental Commons, Biodiversity, and Education

Flanagan et al. identified qualitative themes in the narratives produced by a group of 4th−12th graders in a place-based stewardship education project. The authors focused on two themes of the environmental commons: (1) natural resources and systems on which life depends; and (2) collective actions to protect a community's resources. Specific elements such as awareness of nature, ecological balance, interdependence, environmental identity, generativity, human impact, teamwork, and collective efficacy were discussed in the program.

Fiebelkorn and Menzel explored German and Costa Rican science teachers' estimations of the global distribution of biodiversity and perceptions of the threat to plant species. The authors found that the teachers in both countries recognized Brazil as the country with the highest and most threatened biodiversity, and both groups expressed “spatial optimism” and “overestimation bias.” In addition, the authors found that German teachers had a global perspective of biodiversity loss, while Costa Ricans had a local view.

## Public Perception of Local Environmental Issues

Doran et al. investigated Norwegian and German mental representations in the categorization of different energy transition pathways. The authors found that people from both countries, using card sorting methods, categorized the energy transition components as a multifaceted issue at the individual level (e.g., avoid long flights and cycling), at the societal/political level (taxes and urban development), and at the technological level (e.g., electric cars and solar panels). Cultural differences were observed in the individual level of mental representations.

Böhm et al. further explored Norwegian and German perceptions of energy transition pathways among individual, social/political, and technological levels. The authors employed affective image analysis based on free associations to investigate the affective reaction regarding the issues of energy transition pathways. Participants from both countries showed a similar view of mental images among the three levels.

## Conclusion

Although much progress has been made in understanding value, attitude, motivation, awareness, and behavioral changes resulting from the environmental movement, research on non-WEIRD cultures are underrepresented in the field. This special topic not only assembles cutting edge empirical cultural/cross-cultural research in various topics, but also offers an overview of potential methods and applications of cultural issues in environmental psychology.

## Author Contributions

FJ drafted the manuscript. TK provided feedbacks.

### Conflict of Interest

The authors declare that the research was conducted in the absence of any commercial or financial relationships that could be construed as a potential conflict of interest.
